# Retinal sensitivity and gaze fixation evaluated by microperimetry in subjects with type 2 diabetes: two independent parameters that explore different neuronal circuits

**DOI:** 10.1007/s40618-023-02046-y

**Published:** 2023-03-04

**Authors:** A. M. Ortiz-Zuñiga, A. Rojano Toimil, K. Rahnama, E. Lainez, N. Raguer, O. Simó-Servat, C. Hernández, R. Simó, A. Ciudin

**Affiliations:** 1 Department of Endocrinology and Nutrition, Hospital Universitari Vall Hebron, Pg Vall Hebron 119-129, 08035 Barcelona, Spain; 2 Institut de Recerca Vall d’Hebron, Universitat Autònoma de Barcelona (VHIR-UAB), Pg Vall Hebron 119-129, 08035 Barcelona, Spain; 3 Department of Clinical Neurophysiology, Hospital Universitari Vall Hebron, Pg Vall Hebron 119-129, 08035 Barcelona, Spain; 4 CIBER de Diabetes y Enfermedades Metabólicas Asociadas, Instituto de Salud Carlos III, C/Monforte de Lemos 3-5.Pabellón 11, 28029 Madrid, Spain

**Keywords:** Retinal microperimetry, Retinal sensitivity, Gaze fixation, Type 2 diabetes, Visual evoked potentials

## Abstract

**Background and aims:**

Retinal sensitivity (RS) and gaze fixation (GF) assessed by retinal microperimetry are useful and complementary tools for identifying mild cognitive impairment (MCI) in patients with type 2 diabetes (T2D). The hypothesis is that RS and GF examine different neural circuits: RS depends only on the visual pathway while GF reflects white matter complex connectivity networks. The aim of the study is to shed light to this issue by examining the relationship of these two parameters with visual evoked potentials (VEP), the current gold standard to examine the visual pathway.

**Materials and methods:**

Consecutive T2D patients > 65 years were recruited from the outpatient clinic. Retinal microperimetry (MAIA 3rd generation) and visual evoked potentials (VEP) (Nicolet Viking ED). RS (dB), GF (BCEA63%, BCEA95%) (MAIA) and VEP (Latency P100ms, Amplitude75–100 uV) were analyzed.

**Results:**

Thirty three patients (45% women, 72.1 ± 4.6 years) were included. VEP parameters significantly correlated with RS but not with GF.

**Conclusions:**

These results confirm that RS but not GF depends on the visual pathway, reinforcing the concept that they are complementary diagnostic tools. Used together can further increase the value of microperimetry as screening test for identifying T2D population with cognitive impairment.

## Introduction

Over 50 million people worldwide are affected by dementia, Alzheimer’s disease (AD) being the most common type [1]. Mild cognitive impairment (MCI), is an intermediate stage between normal cognitive function and dementia, which is diagnosed by standard tests and has no significant impact on activities of daily living. MCI affects about 6% of the general population [2], and 20% of the patients > 65 years old [3]. The annual conversion rate from MCI to dementia ranges between 10 and 30% in the general population [4]. Patients with type 2 diabetes (T2D) have about 2.5 higher risk of developing dementia in comparison with age-matched non-diabetic subjects [5]. This increased risk is maintained even after adjusting for vascular risk factors [6, 7]. Moreover, the number of cases of dementia associated with T2D is expected to increase because of the increase in prevalence of diabetic individuals due to a worldwide ageing population.

The American Diabetes Association (ADA) recommends screening for early detection of MCI in patients with T2D older than 65 years [8]. Currently, the diagnosis of cognitive impairment is hampered by an extensive series of complex tests that are difficult to incorporate into daily clinical practice [9].

Retinal microperimetry, is a simple, rapid and non-invasive test that measures retinal sensitivity (RS) in terms of the minimum light intensity that patients can perceive when spots of light stimulate specific areas of the retina, and also evaluates gaze fixation (GF) stability [10, 11]. Our group showed for the first time that the RS significantly correlated with brain imaging (MRI and PET) and identified patients with MCI and dementia, as confirmed by complete and validated neuropsychological battery examination [10].

Furthermore, by adding the GF parameters to the RS the probability to identify cognitive impairment significantly increased in an independent manner, with a sensitivity of 72.7% and sensibility of 87.9% [12], converting the retinal microperimetry into a potential useful tool for the screening of MCI in patients with T2D > 65 years. Additionally, we have recently evaluated the usefulness of retinal microperimetry as a monitoring tool for the cognitive function in patients > 65 years with T2D [13]. The annual worsening in cognitive function went in parallel with worsening of GF, suggesting this parameter can be a reliable and subtler tool for the monitoring of the cognitive function in patients with T2D. Our data suggest that since RS is a reliable screening tool for diagnosis, the evaluation of GF could represent a better biomarker for annual follow-up. These findings could be explained by the hypothesis that RS and GF are examining different neural circuits. Whereas RS depends on the visual pathway, GF reflects the complex white matter connectivity, making them complementary tests in identifying patients with MCI. Visual evoked Potentials (VEP) represents an electrophysiological test used to quantify the functional integrity of the visual pathway and occipital cortex activity, even more sensitive and economical compared to MRI in detecting lesions of the visual pathway [14], and it considered the current gold-standard. Our hypothesis is that RS will correlate with VEP parameters while GF will not. To the best of our knowledge this hypothesis was not explored so far. On these bases we performed the present pilot study aimed at exploring direct correlations between the VEP parameters and retinal microperimetry (RS and GF) in patients with T2D > 65 years with no overt or only mild diabetic retinopathy.

## Materials and methods

Consecutive patients with T2D older than 65 years attended at the outpatient clinic of our hospital between June and December 2019 accomplishing the inclusion criteria were included. The study was conducted according to the Declaration of Helsinki and was approved by the local Ethics Committee (PR(AG)28/2017). Informed consent was obtained from all subjects involved in the study.

The inclusion criteria were: (1) age > 65 years; (2) T2D with a duration > 5 years; (3) no apparent or only mild non-proliferative diabetic retinopathy (DR), according to the Early Treatment Diabetic Retinopathy Study (ETDRS) [15] (4) patients with normal visual acuity (0.8–1). The exclusion criteria were: (1) patients with neurodegenerative diseases of (e.g., Alzheimer’s disease, Parkinson disease, glaucoma, multiple sclerosis); (2) patients with HbA1c > 10% (high blood glucose levels could affect retinal function [16] or hyperglycemic or hypoglycemic decompensations in the last 6 months. (3) Unable or unwilling to perform VEP and retinal microperimetry. (4) Patients with any other ophthalmological disease that could affect the macular integrity.

The presence of diabetic retinopathy was independently evaluated by an ophthalmologist by means of fundoscopy and SD-OCT. Furthermore, during the same visit any fovea or macular alterations were excluded. The visual acuity was evaluated by means of Snellen chart and was quantified as 1.00 (0.0 logMAR) as a criterion for a normal VA in adults. The VA measurement was always performed without optical compensation of the patients, or with their usual optical compensation, in the case of those patients who use glasses or contact lenses. Subjective refraction was not performed to determine the refractive status of the patient, as it was not part of the study protocol.

RS was evaluated by fundus-driven microperimetry (third-generation Macular Integrity Assessment) after pupillary dilation to a minimum of 4 mm and in a dark environment. The standard Macular Integrity Assessment test covers a 10-diameter area with 37 measurement points; a red 1-radius circle was used as the fixation target. A four-level fixed strategy was used: Goldmann III size stimulus, background luminance of 4 asb and maximum luminance of 1000 asb, with a 25-dB dynamic range. We chose this strategy to be in line with our previous studies [10, 12]. Patients were instructed to maintain the gaze fixed on the red radius circle as fixation target and press the control button when on the 37 light stimuli appeared. Notably, the microperimeter automatically compensates for eye movements during examination via a software module that tracks them. The characteristics of fixation (location and stability) were quantified and categorized according to the P1 and P2 parameters, the preferred retinal locus (PRL) used to calculate the fixation stability, as follows: “stable” (P1 and P2 0.75%), “relatively unstable” (P1,75% and P2 0.75%), or unstable (both P1 and P2,75%). The device automatically calculates the reliability index, which assesses the accuracy of the test. Furthermore, according to our previous results [10, 13] we evaluated as part of GF: the bivariate contour ellipse area, as the preferred retinal loci to calculate fixation stability (BCEA): BCEA63% (1 standard deviation) and BCEA95% (2 standard deviations) areas. The BCEA is measured as a bivariate contour ellipse, expressed in “º^2^”.

It should be noted that our study was aimed to evaluate correlations between two functional independent tests (MAIA and VEP) in the same patient and not to test for ophthalmologic disease. We are exploring the potential of MAIA as a reliable tool for cognitive impairment screening and monitoring. For this purpose, in the previous studies we only analyzed the data from the first eye that was evaluated, to avoid “learning-effect” in the second eye. For the same reasons, in the present study we applied the same procedure, including in the analysis data from the right eye in all cased.

Visual evoked potentials (VEP) were recorded in all patients using a Viking Select (Viasys Healthcare, Madison, WI, USA) following the guidelines of the American Clinical Neurophysiology Society [17] and ISCEV recommendations [18]. The visual stimulus was applied using Nicolet 2015 Visual Stimulator (CareFusion NeuroCare, Middleton, WI, USA) with a monitor size of 17 inches in all recordings. Each eye was stimulated separately using a screen full pattern reversal chromatic checks of mid-size (12X16; visual angle = 1° 0′) and small checks (24 × 32; visual angle = 0° 30′). Full-field stimulation was performed monocularly, utilizing a high-contrast (> 50%) black-and-white checkerboard pattern, at a rate of 1.3 cycles per second. The subject was placed 107 cm to the stimulus screen. Visual fixation was at the center of the stimulus screen. Recordings were made using surface electrodes fastened securely over Oz, with Fz as the reference, according to the 10–20 international system of EEG electrode placement. A ground electrode was placed on the forehead. The impedance used in all cases was less than 4000 Ω. The low filter was set at 1 Hz and the high filter at 100 Hz. Recordings were made during the first 500 ms after the stimulus. At least 100 steps were averaged for each tracing, and 2 or 3 replications were run and superimposed to ensure reproducibility. Latency of the P100 component and amplitude N75-P100 were measured for both each eye and each check size. The parameters evaluated for VEP for each check size wer: (1) the presence or absence and latency of wave P100; (2) the N75-P100 amplitude; and (3) symmetry between both eyes. P100 wave is the most robust peak with comparatively minimal interindividual variability, nominal within-subject inter-eye difference, and negligible variation with high repeatability [16]. P100 values from the right eye were used for correlations.

### Sample size calculation

This was a pilot study. Since there is no previous data aimed to explore similar hypothesis, we could not perform sample size calculation.

### Statistical analysis

To assess differences between the groups, a *x*^2^ test was used for qualitative variables and ANOVA followed by a Least Significant Difference (LSD) post hoc test were used for quantitative variables. To evaluate the correlation between VEP and RS and GF variables, a Spearman correlation test and regression analyses adjusted by age were performed. All *p* values were based on a two-tail statistical significance test. Significance was accepted at *p* =  < 0.05. The variables with normal distribution were: age, Body mass index, T2D duration, HbA1C, Retinal microperimetry parameters (Sensitivity, Fixation stability P1 (%), Fixation stability P2 (%), BCEA63% (º^2^), BCEA95% (º^2^)) and Evoked ophthalmic potentials parameters such us Duration (minutes), Latency P100 (ms) 12 × 16; 24 × 34 and Amplitude 75–100 (uV) 12 × 16; 24 × 34. All the correlations were adjusted by gender, age, duration of T2D and smoking status. Statistical analysis was performed with the STATA 15 statistical package.

## Results

A total of 33 patients with T2D (45% women, mean age 72.1 ± 4.6 years) were recruited fulfilling inclusion/exclusion criteria. The characteristics of the patients are shown in Table 1.

**Table 1:** Baseline characteristics of the patients included in the study (*n* = 33)

Clinical characteristics	
Age (years) mean ± SD	72.1 ± 4.6
Gender (women) %	45%
BMI (kg/m^2^) mean ± SD	29.74 ± 0.9
Smoker (%)	59.9%
Hypertension (%)	84.8%
Dyslipidemia (%)	87.9%
Sleep apnea syndrome (%)	16.6%
Ischemic heart disease (%)	18.1%
Peripheral arteriopathy (%)	6.0%
T2D duration (years, mean ± SD)	15.55 ± 7.4
HbA1C (%) (DCCT mean ± SD)	7.38 ± 0.8
Diabetic retinopathy (%)	36.3%
Diabetic nephropathy (%)	27.2%
Diabetic polyneuropathy (%)	18.1%
Visual acuity (1.00)	0.85
Retinal microperimetry (right eye)	
Sensitivity (mean ± SD)	20.75 ± 0.9
Duration (minutes) (mean ± SD)	2.55 ± 0.4
Reliability index % (mean ± SD)	98.1 ± 1.1
Fixation stability P1 (%) (mean ± SD)	70.81 ± 13.3
Fixation stability P2 (%) (mean ± SD)	84.07 ± 4.7
BCEA63%(º^2^) (mean ± SD)	3.67 ± 1.6
BCEA95%(º^2^) (mean ± SD)	32.62 ± 9.3
Evoked ophthalmic potentials (right eye)	
Duration (minutes)	15.16 ± 1.41
Latency P100 (ms)12 × 16 (mean ± SD)	106.43 ± 11.4
Latency P100 (ms)24 × 34 (mean ± SD)	111.97 ± 10.8
Amplitude 75–100 (uV) 12 × 16 (mean ± SD)	6.17 ± 2.8
Amplitude 75–100 (uV) 24 × 34 (mean ± SD)	5.94 ± 2.81

*BMI* body mass index, *T2D* type 2 diabetes, *BCEA63* Bivariate contour ellipse area 63% (º^2^), *BCEA95* Bivariate contour ellipse area 95% (º^2^)

VEP parameters significantly correlated with RS evaluated by microperimetry as reflected by Table 2. By contrast, we did not find any significant correlations between the GF parameters P1 and BCEA95% and VEP, respectively.

**Table 2:** Correlations between microperimetry and VEP parameters

Correlations between microperimetry and VEP	Latency 100 ms	Amplitude (75–100 uV)
Retinal sensitivity (dB)	*R* = 0.716*	*R* = 0.531*
Fixation stability P1 (%)	*R* = 0.175,	*R* = 0.109
Fixation stability P2 (%)	*R* = 0.151	*R* = 0.119
Bivariate contour ellipse area 95% (º^2^)	*R* = 0.156	*R* = 0.238
Bivariate contour ellipse area 63% (º^2^)	*R* = 0.293	*R* = 0.127

*p < 0.05

## Discussion

In the present study we provide first evidence that RS but not GF assessed by microperimetry is correlated with visual pathway explored by VEP in patients with type 2 diabetes, older than 65 years without macular or foveal affectation. As far as we know one study evaluated the relationship between the VEP and microperimetry in patients with macular disease and found correlation between RS and VEP in 48 young (45 years old) patients with altered fixation pattern [19]. The present study provides first data regarding this relationship in subjects without macular disease. Our findings are of interest in the context of proposing the microperimetry a useful tool for screening and monitoring of the cognitive function apart from its current ophthalmologic application [10–12]. In the last years the research from our group focused on demonstrating that retinal microperimetry is useful for screening for cognitive impairment in patients with T2D > 65 years [10, 13]. Furthermore, both parameters, RS and GF when used together increase the ability to correctly identify patients with T2D > 65 years and mild cognitive impairment or dementia, suggesting that they are independent parameters that explore different circuits. Additionally, in a recent study [13], we showed that GF evaluated by microperimetry was useful for the annual monitoring of the cognitive function, while RS remained unchanged in time, supporting the same hypothesis. The results from this pilot study are the first technical piece of evidence towards proving that GF and RS are independent parameters, since RS correlated with the VEP and GF was not. Both parameters, RS and GF can be considered complementary measurements: RS will be more influenced by neurodegeneration while GF by white matter disruption, as we explain further. In the case of RS, the anatomical region involved in the processing of the information that goes through the optic pathway from the retinal ganglion cells, is the lateral geniculate body of the thalamus, which relays information to the primary visual cortex [16]. To support out findings, Arruda et al. [20] has recently suggested the potential role of VEP in detecting amnestic MCI that precedes Alzheimer´s disease, with primary dysfunction of the episodic memory, which is related to neurodegeneration, allowing a connection between the optical pathway and cortical cognitive function. Additionally, RS seems to reflect early alterations in the primary visual cortex. McKee et al. [21] demonstrated the presence of amyloid plaques and neurofibrillary tangles in areas were VEP are generated, before than other areas in the evolution of Alzheimer’s disease. Therefore, it is reasonable that we have found a correlation between RS explored by retinal microperimetry and VEP. We could hypothesize that RS and VEP explore the same neural circuits, nevertheless, future studies could shed light on this interesting findings. In exchange, GF seems to depend on the complex white matter network, the superior colliculus and the parietal and frontal cortex [22]. Additionally, in a previous study published by Ortiz et al. [13], GF but not RS was correlated with attention and delayed recall, which depend mainly on the default brain network and the white matter connections between the dorsolateral and prefrontal cortex and inferior and superior parietal lobules [23, 24]. Furthermore, we also found that GF was impaired in young patients with obesity and significantly correlated with cognitive function, in particular attention and delayed recall, all of them related to white matter network and not visual pathway [12]. Therefore, the present observation that fixation parameters evaluated by retinal microperimetry did not correlate with VEP in our study is not surprising.

As explained in the introduction, the American Diabetes Association recommends annual evaluation of the cognitive function in patients with T2D older than 65 years [8]. Furthermore, it is unfeasible to implement a complete battery of neuropsychological tests for all T2D patients > 65 years because they are time consuming and also depend on the patient´s mood, therefore we urgently need simple and reliable tools. Retinal microperimetry, is a simple, objective and rapid test that can be largely proposed for implementation for the monitoring of cognitive performance, regardless of psychological status or educational level, and can provide two independent and complementary parameters: RS and GF. In this regard, our group is coordinating an ongoing European project (RECOGNISED study, NCT04281186), aimed at validating the usefulness of retinal microperimetry as reliable screening and monitoring tools for cognitive impairment in patients with T2D > 65 years, as well as at exploring its predictive value in the progression of the cognitive decline.

Nevertheless, our study has three main limitations. First, the small sample size can be contemplated as a limiting factor. However, the pilot nature and the design of the study (intra-individual comparisons) makes possible to draw valid results. Second, we have not compared our results with any normative data base. However, it should be noted that normal values of microperimetry and VEP in subjects > 65 years has not been reported. In addition, the aim of the study was not to perform these comparisons but to examine correlations between VEP and either RS and GF to further understand the physiological meaning of these examinations. Third, although the 4 level-fixed strategy used in our study for retinal microperimetry, has large difference of dB between one step and another, we still found significant correlation between RS and VEP.

In conclusion, our results confirm that RS but not GF depends on the visual pathway, reinforcing the concept that they are complementary diagnostic tools. Used together can further increase the value of microperimetry as screening test for identifying T2D population with cognitive impairment. However, further studies are needed in order to validate our results in larger cohorts of patients and to deepen the underlying circuits and mechanisms involved.

## Data Availability

Data is available on request to the corresponding authors.
